# Timing of rehabilitation initiation and rehabilitation duration, and related factors in occupational injuries: a single-center retrospective analysis

**DOI:** 10.3389/fpubh.2026.1743735

**Published:** 2026-04-23

**Authors:** Lan Zhu, Rao Xu, Yuanyue Chen, Jun Zhu, Zhixiang Wang, Chuan Guo, Jingjun Hu

**Affiliations:** 1 Department of Rehabilitation Medicine, Nanjing Qixia District Hospital, Nanjing, China; 2 Department of Rehabilitation Medicine, The First Affiliated Hospital With Nanjing Medical University, Nanjing, China; 3 Department of Rehabilitation Medicine, Jiangsu Province (Suqian) Hospital, Suqian, Jiangsu, China; 4 Department of Rehabilitation Medicine, Yancheng Dexin Hospital, Yancheng, China; 5 Department of Rehabilitation Medicine, Yancheng Ruikang Hospital, Yancheng, China

**Keywords:** duration of rehabilitation, occupational injuries, occupational rehabilitation, rehabilitation costs, retrospective analysis, timing of rehabilitation initiation

## Abstract

**Objective:**

To retrospectively analyze the timing of rehabilitation initiation and the duration of rehabilitation among patients with occupational injuries at a designated rehabilitation hospital in Yancheng, China, and to identify the influencing factors. The findings aim to provide valuable insights to support the development of China’s occupational rehabilitation service system.

**Methods:**

A retrospective analysis was conducted on the medical records of workers with occupational injuries who received rehabilitation treatment at a designated occupational rehabilitation hospital in Yancheng from 2021 to 2025. Quantile regression analysis was employed to investigate the factors influencing the timing of rehabilitation initiation and the duration of rehabilitation.

**Results:**

No statistically significant differences were observed in the timing of rehabilitation initiation concerning sex, age group, injury site, surgical status, rehabilitation costs, or the duration of rehabilitation (*p* > 0.05), except for the administrative origin of injury certification (*p* < 0.001). In contrast, the duration of rehabilitation was significantly associated with all examined variables, including male sex, the age group of 60–69 years, injury site, surgical intervention, administrative origin of injury certification, and rehabilitation costs (*p* < 0.05).

**Conclusion:**

The timing of rehabilitation intervention for workers with occupational injuries depends on the administrative origin of injury certification. Meanwhile, the duration of rehabilitation is closely linked to factors, including sex, age group, injury site, surgical intervention, the administrative origin of injury certification, and rehabilitation costs. This study offers valuable evidence for optimizing occupational rehabilitation policies and services in Yancheng, China.

## 1 Introduction

With the rapid growth of industrialization and urbanization, occupational accidents have been steadily rising, posing a threat to the health and safety of employees, increasing the economic burdens of employers, and impacting social stability and public health. To a certain extent, these factors hinder the development of social productivity (1). Data from the World Health Organization (WHO) and the International Labour Organization (ILO) indicate that approximately 2 million people die each year due to occupational injuries (2). According to estimates, occupational accidents account for about 4% of the global gross domestic product (GDP) (3). This clearly demonstrates that occupational injuries have become a critical global challenge in public health and labor protection.

Occupational rehabilitation consists of coordinated and standardized medical, vocational, and social rehabilitation services for workers with occupational injuries. These services aim to restore physical function, rebuild work capacity, and support workers in returning to employment and social participation. Occupational rehabilitation plays a central role in the integrated prevention–rehabilitation–compensation system (where “compensation” refers to financial benefits provided to workers after occupational injury certification) and forms the foundation of occupational injury insurance. It addresses the limitations of traditional models that overemphasize financial compensation and reflects a transition toward a function-oriented rehabilitation framework (4, 5). Effective occupational rehabilitation contributes to functional recovery and quality of life while enhancing system efficiency and sustainability at the macro level by shortening treatment duration and reducing compensated work absence (6). At the macro level, occupational rehabilitation can shorten the duration of medical treatment and compensated work absence. It helps reduce medical and compensation costs and improves the sustainability of the insurance system. Data and risk assessment information collected during rehabilitation offer essential support for injury grading, workplace safety management, and occupational injury prevention policies (7, 8). These measures together help establish a closed-loop mechanism linking prevention, injury, rehabilitation, and re-prevention (9, 10).

Internationally, occupational rehabilitation has become a vital part of occupational injury insurance systems. Developed countries prioritize rehabilitation over compensation to improve recovery outcomes through structured systems and optimized service delivery. For example, Germany upholds the principle of rehabilitation before compensation. This principle is protected by national legislation, and a structured, standardized, and phased rehabilitation system has been established to ensure effective coordination between medical and vocational rehabilitation (11). The United States relies on market-based mechanisms, emphasizing early intervention, case management, and the integration of community resources. The system focuses on individualized rehabilitation pathways and adopts a return-to-work–oriented approach to support functional and vocational rehabilitation (12, 13). Building on these experiences, strategies such as early rehabilitation intervention, intersectoral collaboration, and a return-to-work orientation have been identified as key approaches to further improve rehabilitation efficiency and support the development of a standardized and effective service system.

In contrast, the development of occupational rehabilitation in China started relatively late, and the institutional framework remains under development. Practical challenges include regional disparities, uneven resource allocation, and a lack of standardized services (14). However, recently, the Chinese government has placed greater importance on occupational rehabilitation, with strengthened policy support. In 2007, the Ministry of Human Resources and Social Security launched pilot programs for occupational rehabilitation. In 2013, the Trial Catalogue of Occupational Rehabilitation Services was introduced, defining the scope and standards of rehabilitation services. Notably, in 2023, seven national ministries jointly issued the Guidelines on Promoting the High-Quality Development of Occupational Rehabilitation, which outlined a comprehensive plan to improve the rehabilitation service system toward greater standardization and institutionalization.

Against this policy backdrop, this study takes Yancheng, China, as a research case example and utilizes medical data from designated occupational rehabilitation hospitals from 2021 to 2025. It systematically analyzes the key features of the timing of rehabilitation initiation and its duration among workers with occupational injuries, exploring their related and distinct influencing factors. The aim is to identify major bottlenecks in local rehabilitation service practices and provide empirical evidence to support policies and optimize service processes. Previous studies have demonstrated that early rehabilitation intervention facilitates functional recovery, shortens the rehabilitation period, and reduces the economic burden (15). Therefore, this study aims to provide insights for improving the occupational rehabilitation system and enhancing service quality in Yancheng, while also contributing practical experience for developing high-quality occupational rehabilitation policies in China.

## 2 Methods

### 2.1 General information

Medical data were collected from workers who were occupationally injured and received rehabilitation training at Yancheng Dexin Hospital between January 2021 and June 2025. As the designated occupational rehabilitation center in Yancheng, the hospital provides clinical data extracted from its official medical records system. Variables included sex, age group, injury site, surgical status, the administrative origin of injury certification, rehabilitation costs, the timing of rehabilitation initiation, and the duration of rehabilitation. Inclusion criteria: patients with a valid administrative origin of injury certification issued by the Yancheng Municipal Human Resources and Social Security Bureau. Exclusion criteria: cases without occupational injury certification or with incomplete or inaccurate data that could not be reliably corrected.

A total of 897 cases of workers who were occupationally injured were finally included in the study. The study protocol was approved by the Ethics Committee of The First Affiliated Hospital with Nanjing Medical University (Approval No. 2023-SR-311). The committee waived the requirement for informed consent, as the data were retrospectively collected and anonymized. This waiver is in accordance with Article 32 of the *Measures for the Ethical Review of Biomedical Research Involving Humans* (China, 2023).

### 2.2 Study design

The medical data of workers with occupational injuries were extracted from the hospital’s electronic medical records system and entered into an Excel spreadsheet by a trained data manager. An independent data manager verified the dataset to ensure completeness and accuracy. Descriptive analyses were performed to summarize general variables, including sex, age, injury site, surgical status, and the administrative origin of injury certification. Based on these analyses, relevant factors influencing the timing of rehabilitation initiation and the duration of rehabilitation were subsequently examined.

### 2.3 Statistical analysis

Data were analyzed statistically using SPSS software (version 26.0; IBM Corp, Chicago, United States). Categorical variables were described using frequencies and proportions. Continuous variables did not follow a normal distribution, as determined by normality testing; therefore, they were expressed as the median (M) and interquartile range, expressed as the 25th and 75th percentiles (*P*_25_, *P*_75_). The Mann–Whitney *U* test was used to compare two groups, while the Kruskal–Wallis *H* test was used for comparisons among multiple groups. *Post hoc* pairwise comparisons were performed with the Bonferroni-adjusted multiple testing procedure. A two-sided *p* < 0.05 was considered statistically significant. The timing of rehabilitation initiation and the duration of rehabilitation were not normally distributed. To explore factors associated with them, quantile regression models were constructed using these two variables as dependent outcomes, respectively, with the other variables as independent predictors. Quantile regression analyses were performed at the 0.25, 0.50 (median), and 0.75 quantiles of the dependent variable to assess the potential heterogeneity of associations across its distribution. Prior to modeling, multicollinearity among the independent variables was assessed using the Variance Inflation Factor (VIF), with VIF values below 5 indicating no significant multicollinearity. Model performance was evaluated using the pseudo *R*^2^ statistic, indicating how effectively the model accounts for variations in the dependent variable.

## 3 Results

### 3.1 Comparison of baseline characteristics

Of the 897 workers with occupational injuries who received rehabilitation from 2021 to 2025, the median timing of rehabilitation initiation was 126 days, and the median duration of rehabilitation was 30 days.

**Figure 1:**
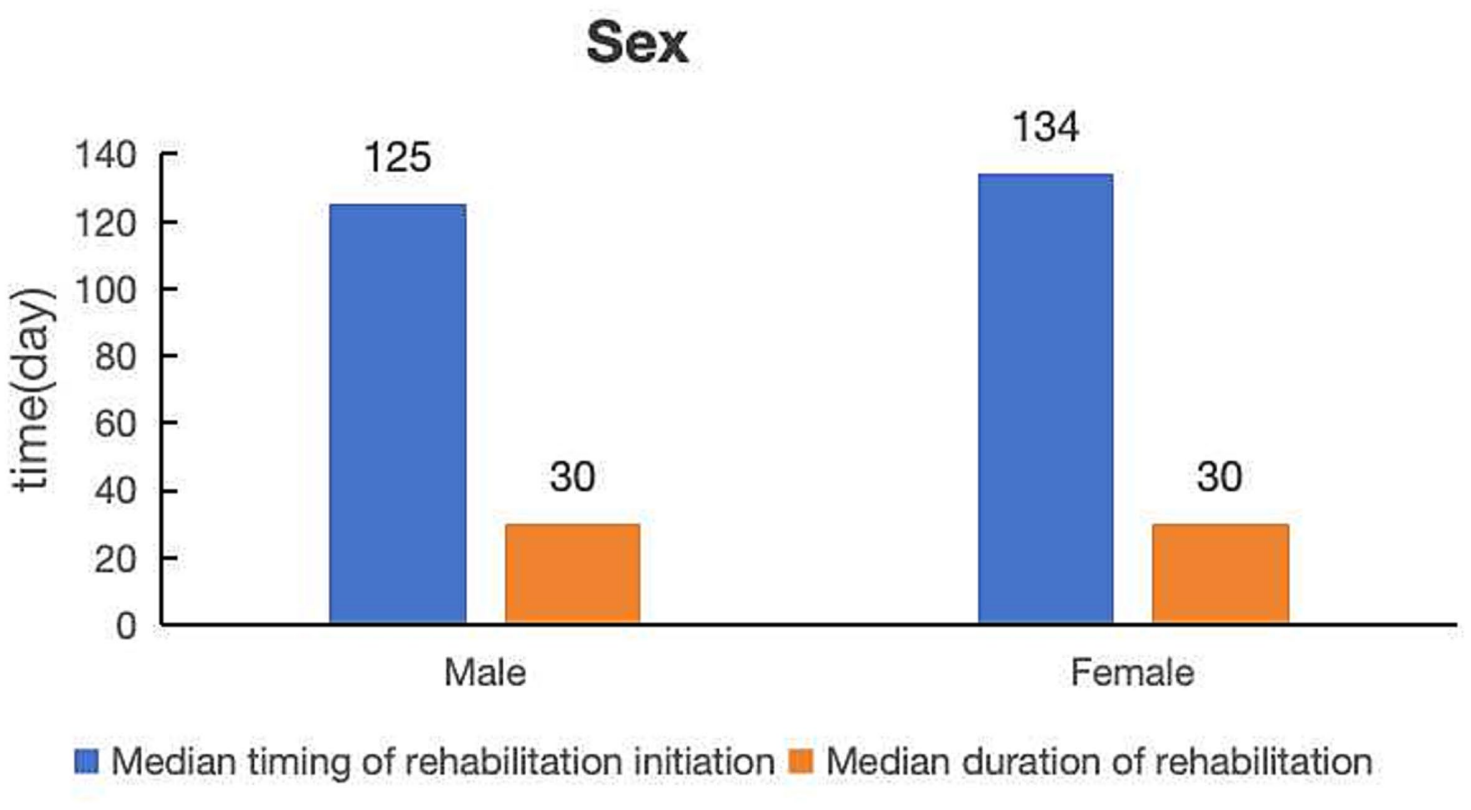
Comparison of median timing of rehabilitation initiation and median duration of rehabilitation by sex.

Most workers with occupational injuries were male (*n* = 668, 74.47%) (Table 1), with a median timing of rehabilitation initiation of 125 days and a median duration of rehabilitation of 30 days (Figure 1).

**Table 1:** Timing of rehabilitation initiation by different demographic and clinical characteristics.

Variable	Frequency (percentage)	*M* (*P*_25_, *P*_75_)	*H/Z-value*	*p*
Sex			−0.997	0.319
Male	668 (74.47%)	125 (80, 216)		
Female	229 (25.53%)	134 (81, 232)		
Age group			7.488	0.112
20–29 years	77 (8.58%)	146 (97, 224)		
30–39 years	227 (25.31%)	115 (75, 214)		
40–49 years	288 (32.11%)	125 (80, 210)		
50–59 years	285 (31.77%)	134 (85, 236)		
60–69 years	20 (2.23%)	162 (97, 300)		
Injury site			15.247	0.018*
Head and neck injuries	26 (2.90%)	130 (83, 278)		
Upper limb injuries	147 (16.39%)	133 (85, 239)		
Trunk injuries	53 (5.91%)	125 (83, 202) i		
Lower limb injuries	314 (35.01%)	130 (88, 241)		
Hand injuries	293 (32.66%)	115 (75, 181)		
Burn	15 (1.67%)	150 (77, 262)		
Multiple injuries	49 (5.46%)	158 (87, 283)		
Surgical status			−0.368	0.713
Yes	757 (84.39%)	127 (81, 223)		
No	140 (15.61%)	120 (83, 215)		
Administrative origin of injury certification			190.223	<0.001*
Yancheng (Central)	72 (8.03%)	100 (70, 151) srw		
Yandu	94 (10.48%)	118 (94, 181) sxv		
Tinghu	67 (7.47%)	119 (86, 232) sxv		
Chengnan	30 (3.34%)	115 (70, 164) s		
Kaifaqu	58 (6.47%)	98 (60, 168) srw		
Dafeng	139 (15.50%)	147 (106, 249) s		
Dongtai	98 (10.92%)	248 (204, 330)		
Jianhu	31 (3.46%)	85 (65, 150) sr		
Funing	38 (4.24%)	130 (73, 287) s		
Binhai	86 (9.59%)	83 (67, 137) srw		
Sheyang	104 (11.59%)	152 (103, 230) s		
Xiangshui	80 (8.92%)	89 (57, 133) srw		

For age groups, statistical significance at *p* < 0.05 is denoted as follows:

a = compared with 20–29 years; b = compared with 30–39 years; c = compared with 40–49 years; d = compared with 50–59 years; e = compared with 60–69 years.

For injury sites, statistical significance at *p* < 0.05 is denoted as:

f = compared with head and neck injuries; g = compared with upper limb injuries; h = compared with trunk injuries; i = compared with lower limb injuries; j = compared with hand injuries; k = compared with burns; l = compared with multiple injuries.

For the administrative origin of injury certification, statistical significance at *p* < 0.05 is denoted as:

m = compared with Yancheng; n = compared with Yandu; o = compared with Tinghu; p = compared with Chengnan; q = compared with Kaifaqu; r = compared with Dafeng; s = compared with Dongtai; t = compared with Jianhu; u = compared with Funing; v = compared with Binhai; w = compared with Sheyang; x = compared with Xiangshui.

*p* < 0.05 was considered statistically significant. Asterisks (*) denote statistically significant differences.

**Figure 2:**
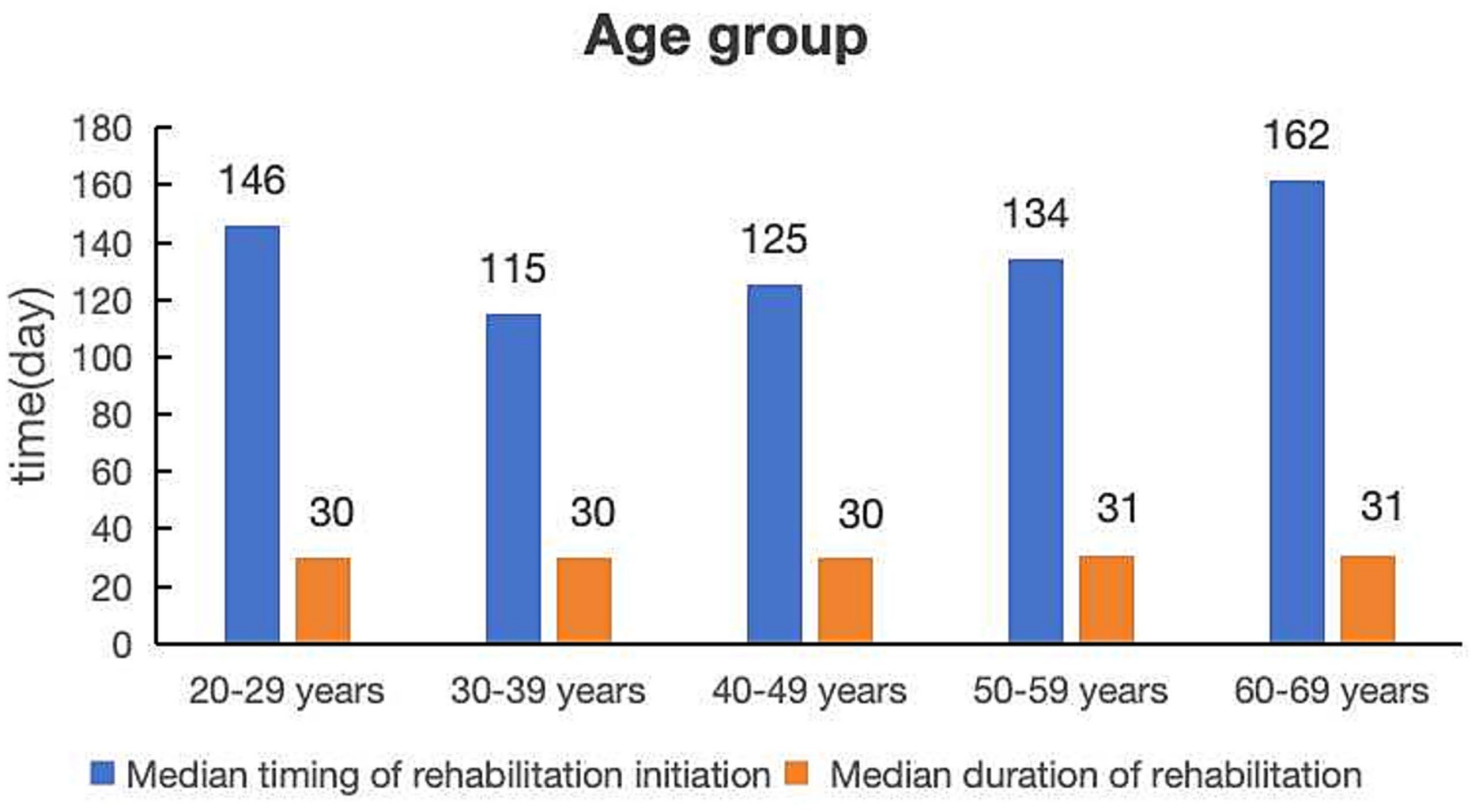
Comparison of median timing of rehabilitation initiation and median duration of rehabilitation by age group.

In terms of age groups, the largest proportion of workers receiving rehabilitation fell within the 40–49 age group (*n* = 288, 32.10%; Table 1), with a median timing of rehabilitation initiation of 125 days and a median duration of rehabilitation of 30 days (Figure 2). In contrast, the smallest proportion was observed among those aged 60–69 years (*n* = 20, 2.23%), who had the longest median timing of rehabilitation initiation (162 days) and the longer duration of rehabilitation (31 days).

**Figure 3:**
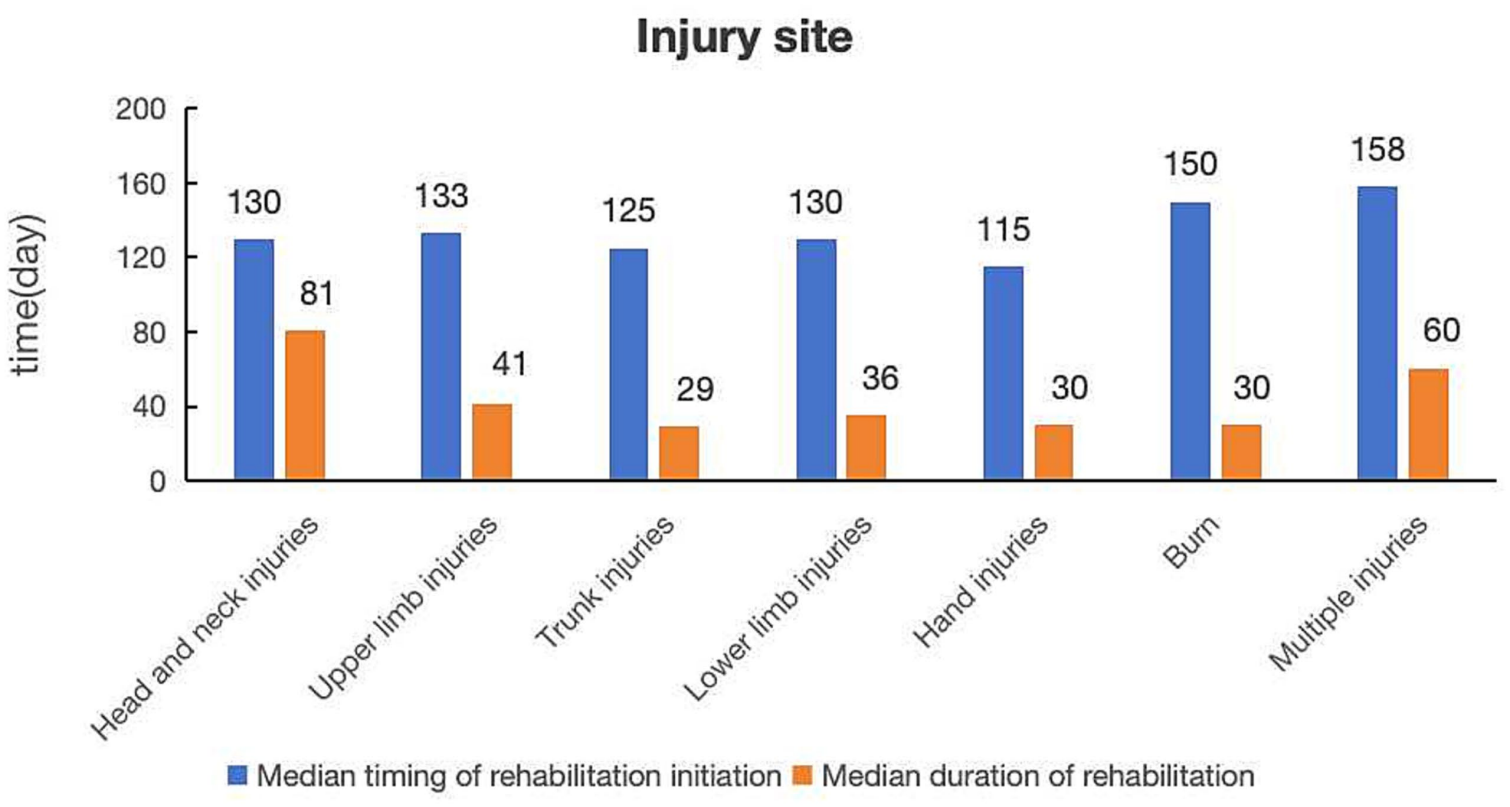
Comparison of median timing of rehabilitation initiation and median duration of rehabilitation by injury site.

Regarding injury site, lower limb and hand injuries were the most common, accounting for 314 (35.01%) and 293 (32.27%) cases, respectively (Table 1). The median timing of rehabilitation initiation in these groups were 130 and 115 days, with corresponding median duration of rehabilitation of 36 and 30 days (Figure 3). In contrast, burns (*n* = 15, 1.67%), head and neck injuries (*n* = 26, 2.90%), and multiple-site injuries (*n* = 49, 5.46%) were relatively uncommon. Their median timing of rehabilitation initiation was longer, at 150, 130, and 158 days, respectively, while the median durations of rehabilitation were 30, 80, and 60 days (Table 1; Figure 3).

**Figure 4:**
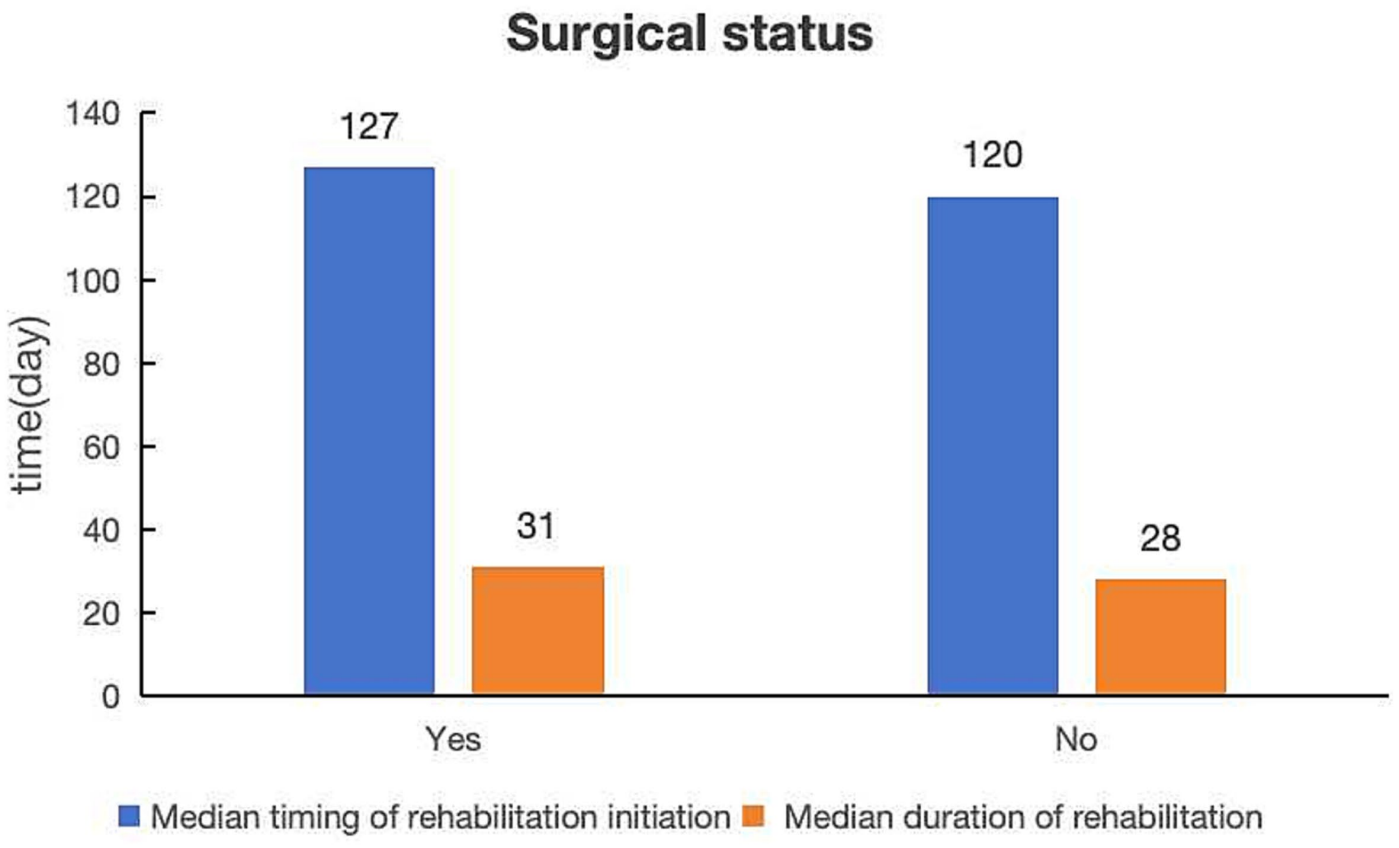
Comparison of median timing of rehabilitation initiation and median duration of rehabilitation by surgical status.

Regarding surgical status, 757 workers with occupational injuries (84.43%) underwent surgery (Table 1), with a median timing of rehabilitation initiation of 127 days and a median duration of rehabilitation of 31 days (Figure 4). Meanwhile, the non-surgical worker-injury employees (*n* = 140, 15.61%) had a slightly shorter timing of rehabilitation initiation (120 days) and a markedly shorter duration of rehabilitation (28 days).

**Figure 5:**
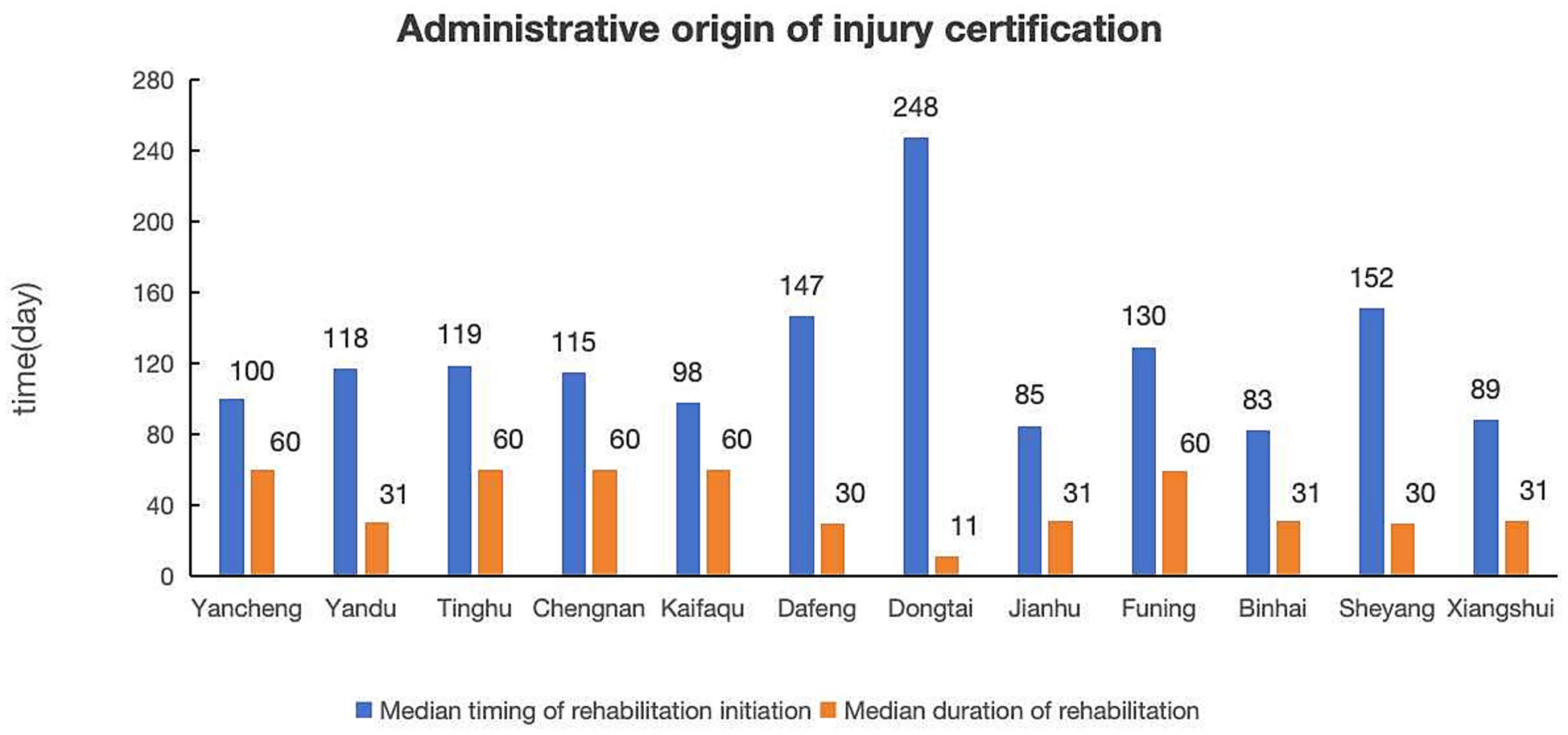
Comparison of median timing of rehabilitation initiation and median duration of rehabilitation by administrative origin of injury certification.

Based on the administrative origins of injury certification, there are 12 jurisdictions in Yancheng. Dafeng had the highest representation (*n* = 139, 15.50%) (Table 1), with a median timing of rehabilitation initiation of 147 days and a duration of rehabilitation of 30 days (Figure 5). Dongtai ranked second in representation (*n* = 98, 10.92%). It had the longest timing of rehabilitation initiation (248 days), but the shortest duration of rehabilitation (11 days). Although Funing had fewer participants (*n* = 38, 4.24%), it indicated a median timing of rehabilitation initiation of 130 days and a median duration of rehabilitation of 60 days.

### 3.2 The timing of rehabilitation initiation and the duration of rehabilitation across baseline characteristics

A comparative analysis of the timing of rehabilitation initiation across various demographic and clinical factors revealed no statistically significant differences based on sex, age group, or surgical status (*p* > 0.05). However, injury site (*p* < 0.05) and the administrative origin of injury certification (*p* < 0.001) were significantly associated with the timing of rehabilitation initiation (Table 1).

Regarding the duration of rehabilitation, no significant differences were observed between groups based on sex or age group (*p* > 0.05). In contrast, the duration of rehabilitation was significantly influenced by injury site, surgical status, and the administrative origin of injury certification (*p* < 0.001) (Table 2).

**Table 2:** Duration of rehabilitation by different demographic and clinical characteristics.

Variable	Frequency (percentage)	*M* (*P*_25_, *P*_75_)	*H/Z*-value	*p*
Sex			−0.417	0.677
Male	668 (74.47%)	30 (14, 61)		
Female	229 (25.53%)	30 (27, 60)		
Age group			7.579	0.108
20–29 years	77 (8.58%)	30 (11, 60)		
30–39 years	227 (25.31%)	30 (15, 88)		
40–49 years	288 (32.11%)	30 (15, 60)		
50–59 years	285 (31.77%)	31 (14, 61)		
60–69 years	20 (2.23%)	31 (29, 88)		
Injury site			48.652	<0.001*
Head and neck injuries	26 (2.90%)	81 (26, 181) ig		
Upper limb injuries	147 (16.39%)	41 (19, 88) i		
Trunk injuries	53 (5.91%)	29 (14, 60)		
Lower limb injuries	314 (35.01%)	36 (24, 61) i		
Hand injuries	293 (32.66%)	30 (13, 60)		
Burn	15 (1.67%)	30 (14, 32)		
Multiple injuries	49 (5.46%)	60 (29, 90) i		
Surgical status			−6.333	<0.001*
Yes	757 (84.39%)	31 (17, 72)		
No	140 (15.61%)	28 (10, 31)		
Administrative origin of injury certification			88.964	<0.001*
Yancheng (Central)	72 (8.03%)	60 (30, 61) s		
Yandu	94 (10.48%)	31 (15, 60) s		
Tinghu	67 (7.47%)	60 (30, 90) swr		
Chengnan	30 (3.34%)	60 (30, 90) s		
Kaifaqu	58 (6.47%)	60 (29, 90) s		
Dafeng	139 (15.50%)	30 (14, 60) s		
Dongtai	98 (10.92%)	11 (10, 30)		
Jianhu	31 (3.46%)	31 (29, 91) s		
Funing	38 (4.24%)	60 (29, 90) sw		
Binhai	86 (9.59%)	31 (14, 70) s		
Sheyang	104 (11.59%)	30 (14, 60) s		
Xiangshui	80 (8.92%)	31 (15, 83)s		

For age groups, statistical significance at *p* < 0.05 is denoted as follows:

a = compared with 20–29 years; b = compared with 30–39 years; c = compared with 40–49 years; d = compared with 50–59 years; e = compared with 60–69 years.

For injury sites, statistical significance at p < 0.05 is denoted as:

f = compared with head and neck injuries; g = compared with upper limb injuries; h = compared with trunk injuries; i = compared with lower limb injuries; j = compared with hand injuries; k = compared with burns; l = compared with multiple injuries.

For the administrative origin of injury certification, statistical significance at *p* < 0.05 is denoted as:

m = compared with Yancheng; n = compared with Yandu; o = compared with Tinghu; p = compared with Chengnan; q = compared with Kaifaqu; r = compared with Dafeng; s = compared with Dongtai; t = compared with Jianhu; u = compared with Funing; v = compared with Binhai; w = compared with Sheyang; x = compared with Xiangshui.

*p* < 0.05 was considered statistically significant. Asterisks (*) denote statistically significant differences.

### 3.3 Quantile regression analysis of factors influencing the timing of rehabilitation initiation

Before model construction, multicollinearity among independent variables was assessed using the variance inflation factor (VIF). The VIF values ranged from 1.088 to 7.364. “Lower limb injury” (VIF = 7.364) and “hand injury” (VIF = 6.504) slightly exceeded the predefined threshold of 5, whereas all other variables were below this value. However, all VIFs remained below 10, suggesting the absence of serious multicollinearity. Given their strong clinical relevance as key injury site categories, these variables were retained in the analysis. Therefore, all variables were included in the quantile regression models. The timing of rehabilitation initiation was set as the dependent variable, and quantile regression models were constructed at the 25th, 50th, and 75th percentiles. The corresponding pseudo *R*^2^ values were 0.103, 0.119, and 0.104, respectively, indicating a modest but consistent explanatory capacity across different quantiles.

Only the administrative origin of injury certification was significantly associated with the timing of rehabilitation initiation (*p* < 0.05). Specifically, Dafeng, Dongtai, and Sheyang demonstrated consistent significance across multiple quantiles, whereas Xiangshui showed significant effects predominantly at lower quantiles, and Tinghu only at higher quantiles. In addition, trunk injuries and hand injuries also had significant associations with the timing of rehabilitation initiation. Other variables, including sex, age group, site of injury, surgical status, the duration of rehabilitation, and rehabilitation medical expenses, showed no significant associations (*p* > 0.05). Detailed results are shown in Table 3.

**Table 3:** Quantile regression analysis of factors influencing the timing of rehabilitation initiation.

Category	Variable	*P*_25_			*P*_50_			*P*_75_		
		Coefficient	*t*	*p*-value	Coefficient	*t*	*p*-value	Coefficient	*t*	*p*-value
Sex	Female (Ref)									
	Male	−6.848	−1.674	0.094	−12.838	−1.824	0.068	−5.019	−0.289	0.773
Age group	20–29 years (Ref)									
	30–39 years	−12.887	−1.784	0.075	−11.757	−0.946	0.344	−25.772	−0.839	0.402
	40–49 years	−2.992	−0.839	0.402	−5.070	−0.826	0.409	−13.441	−0.886	0.376
	50–59 years	−1.339	−0.565	0.572	−2.537	−0.623	0.533	−9.294	−0.923	0.356
	60–69 years	3.350	1.188	0.235	9.448	1.948	0.052	10.168	0.848	0.397
Injury site	Head and injuries (Ref)									
	Upper limb injuries	0.313	0.030	0.976	1.593	0.089	0.929	−65.732	−1.481	0.139
	Trunk injuries	−3.391	−0.653	0.514	−10.072	−1.128	0.260	−50.681	−2.295	0.022 *
	Lower limb injuries	2.085	0.645	0.519	1.262	0.227	0.820	−23.117	−1.682	0.093
	Hand injuries	0.221	0.088	0.930	−3.767	−0.875	0.382	−27.599	−2.593	0.010*
	Burn	−0.629	−0.268	0.789	−3.871	−0.959	0.338	−16.823	−1.685	0.092
	Multiple injuries	3.553	1.755	0.080	2.383	0.685	0.494	−10.167	−1.181	0.238
Surgical status	No (Ref)									
	Yes	5.883	1.204	0.229	8.464	1.007	0.314	23.959	1.154	0.249
Administrative origin of injury certification	Yancheng (Ref)									
	Yandu	14.338	1.790	0.074	33.240	2.412	0.016*	48.288	1.417	0.157
	Tinghu	6.010	1.416	0.157	12.797	1.753	0.080	35.711	1.979	0.048*
	Chengnan	6.865	1.958	0.051	15.167	2.514	0.012 *	23.416	1.570	0.117
	Kaifaqu	−5.379	−2.145	0.032*	0.382	0.089	0.929	8.355	0.784	0.434
	Dafeng	6.773	4.620	<0.001*	14.794	5.865	<0.001*	24.704	3.962	<0.001*
	Dongtai	17.027	12.910	<0.001*	24.920	10.984	<0.001*	34.893	6.222	<0.001*
	Jianhu	−1.614	−1.190	0.235	−0.289	−0.124	0.902	1.091	0.189	0.850
	Funing	−0.632	−0.450	0.653	0.110	0.046	0.964	9.154	1.532	0.126
	Binhai	−1.050	−1.140	0.254	−0.351	−0.222	0.825	0.095	0.024	0.981
	Sheyang	2.541	3.262	0.001*	5.482	4.092	<0.001*	8.957	2.704	0.007 *
	Xiangshui	−1.925	−2.537	0.011*	−0.590	−0.452	0.651	0.099	0.031	0.976
Rehabilitation cost		0.000	1.311	0.190	0.000	0.474	0.636	0.000	−0.445	0.657
Rehabilitation duration		−0.040	−0.672	0.502	−0.024	−0.234	0.815	0.130	0.510	0.610

*p* < 0.05 was considered statistically significant. Asterisks (*) denote statistically significant differences. Ref indicates the reference category.

### 3.4 Quantile regression analysis of factors influencing the duration of rehabilitation

Multicollinearity among independent variables was assessed using the variance inflation factor (VIF). The VIF values ranged from 1.060 to 7.299. “Lower limb injury” (VIF = 7.299) and “hand injury” (VIF = 6.382) slightly exceeded the predefined threshold of 5, whereas all other variables were below this value. However, all VIFs remained below 10, and thus all variables were retained in the analysis. Using the duration of rehabilitation as the dependent variable, quantile regression models were constructed at the 25th, 50th, and 75th quantiles. The corresponding pseudo *R*^2^ values were 0.536, 0.655, and 0.713, respectively, indicating good and stable model fit across different quantiles.

Quantile regression analysis with the duration of rehabilitation as the dependent variable revealed that sex, age group, injury site, surgical status, the administrative origin of injury certification, and rehabilitation medical expenses were significantly associated with the duration of rehabilitation (*p* < 0.05). Male workers had significantly shorter durations of rehabilitation than females at the 25th and 50th quantiles. Participants aged 60–69 years had significantly shorter durations of rehabilitation across all quantiles (*p* < 0.05). Regarding injury site, upper limb, lower limb, hand trauma, and trunk injuries significantly affected the duration of rehabilitation at the lower and median quantiles. Surgical cases demonstrated significantly longer durations of rehabilitation across all quantiles (*p* < 0.05). Significant differences in rehabilitation duration were observed across localities with work injury certification, with Yandu, Dafeng, Dongtai, Binhai, and Sheyang showing effects at multiple quantiles. Rehabilitation medical expenses were positively associated with the duration of rehabilitation across all quantiles (*p* < 0.001), whereas the timing of rehabilitation initiation was not significantly associated (*p* > 0.05). Detailed results are available in Table 4.

**Table 4:** Quantile regression analysis of factors influencing the duration of rehabilitation.

Category	Variable	*P_25_*			*P_50_*			*P_75_*		
		Coefficient	*t*	*p*-value	Coefficient	*t*	*p*-value	Coefficient	*t*	*p*-value
Sex	Female (Ref)									
	Male	−2.128	−4.157	<0.001*	−2.332	−3.470	<0.001*	−1.563	−1.795	0.073
Age group	20–29 years (Ref)									
	30–39 years	−0.409	−0.453	0.651	−1.746	−1.472	0.141	−1.271	−0.827	0.408
	40–49 years	−0.322	−0.722	0.470	−0.314	−0.536	0.592	0.279	0.368	0.713
	50–59 years	−0.066	−0.225	0.822	−0.467	−1.200	0.230	−0.214	−0.426	0.670
	60–69 years	−0.795	−2.253	0.024*	−1.327	−2.865	0.004*	−1.375	−2.293	0.022*
Injury site	Head and neck injuries (Ref)									
	Upper limb injuries	28.635	22.009	<0.001*	11.579	6.778	<0.001*	7.524	3.400	<0.001*
	Trunk injuries	11.912	18.489	<0.001*	2.612	3.087	0.002*	0.391	−0.356	0.722
	Lower limb injuries	8.930	22.185	0.001*	2.939	5.560	<0.001*	1.457	2.128	0.034*
	Hand injuries	6.407	20.656	0.001*	1.989	4.883	<0.001*	0.992	1.881	0.060
	Burn	4.694	16.054	0.001*	0.856	2.230	0.026*	−0.097	−0.194	0.846
	Multiple injuries	4.393	17.364	0.001*	1.301	3.918	<0.001*	0.474	1.101	0.271
Surgical status	No (Ref)									
	Yes	1.890	3.134	0.002*	1.751	2.212	0.027*	2.244	2.188	0.029*
Administrative origin of injury certification	Yancheng (Ref)									
	Yandu	−4.365	−4.351	<0.001*	−8.013	−6.084	<0.001*	−10.853	−6.36	<0.001*
	Tinghu	−0.333	−0.629	0.530	−1.675	−2.406	0.016*	−1.195	−1.326	0.185
	Chengnan	−0.395	−0.898	0.369	−0.541	−0.937	0.349	−1.014	−1.356	0.175
	Kaifaqu	−0.209	−0.667	0.505	−1.035	−2.512	0.012*	−1.009	−1.890	0.059
	Dafeng	−0.644	−3.487	<0.001*	−1.313	−5.415	<0.001*	−1.856	−5.908	<0.001*
	Dongtai	−1.075	−6.319	<0.001*	−1.738	−7.782	<0.001*	−2.371	−8.195	<0.001*
	Jianhu	−0.240	−1.417	0.157	−0.590	−2.650	0.008	−1.026	−3.556	<0.001*
	Funing	−0.082	−0.468	0.640	−0.172	−0.746	0.456	−0.006	−0.019	0.985
	Binhai	−0.463	−4.018	<0.001*	−0.673	−4.448	<0.001*	−0.977	−4.987	<0.001*
	Sheyang	−0.474	−4.855	<0.001*	−0.814	−6.347	<0.001*	−1.272	−7.660	<0.001*
	Xiangshui	−0.227	−2.396	0.017	−0.396	−3.177	<0.001*	−0.754	−4.671	<0.001*
Rehabilitation cost		0.002	254.325	<0.001*	0.002	219.885	<0.001*	0.002	192.257	<0.001*
Rehabilitation initiation time		0.001	0.731	0.465	0.002	0.928	0.353	0.005	1.790	0.074

*p* < 0.05 was considered statistically significant. Asterisks (*) denote statistically significant differences. Ref indicates the reference category.

## 4 Discussion

This study investigated occupational rehabilitation among workers with occupational injuries in Yancheng from 2021 to 2025. It systematically examined the factors influencing both the timing of rehabilitation initiation and the duration of rehabilitation. The results indicated that significant differences existed in both the timing of rehabilitation initiation and the duration of rehabilitation across workers from different administrative origins of injury certification, suggesting that this factor may play an important role in the rehabilitation process among workers with occupational injuries. These findings indicate that there was an imbalance in the implementation of occupational rehabilitation policies among different administrative regions in Yancheng. Specifically, workers with occupational injuries in Dongtai, Dafeng, and Tinghu showed significantly prolonged the timing of rehabilitation initiation at the 25th, 50th, and 75th percentiles. In contrast, in Xiangshui, a significant prolongation was observed only at the 75th percentile. Similarly, the duration of rehabilitation was significantly extended in multiple quantiles for workers with occupational injuries in Yandu, Dafeng, Dongtai, Binhai, and Sheyang.

Notably, taking specific regions as an example, the median timing of rehabilitation initiation for workers with occupational injuries in Dongtai exceeded 240 days, while the median duration of rehabilitation was the shortest at only 11 days. This marked discrepancy suggests substantial regional variation in rehabilitation pathways. Although a definitive causal relationship cannot be established in this study, these findings may be partly associated with the non-standardized and potentially complex referral processes in the region, including multiple administrative steps and delays in application processing. Such variations in referral practices may influence both the timing of access to rehabilitation services and the characteristics of patients ultimately admitted for rehabilitation. For instance, stricter screening or delayed referral may result in a higher proportion of more severe cases being admitted (16). The regional disparities observed in this study underscore the need for a standardized and efficient rehabilitation referral system (17). Establishing such a system may promote equitable access to rehabilitation services and improve the overall quality of occupational rehabilitation in Yancheng (18).

This study found that rehabilitation costs were not significantly associated with the timing of rehabilitation initiation among workers with occupational injuries, although they were positively associated with the duration of rehabilitation, consistent with previous studies. The lack of association with initiation timing may reflect regional differences in the implementation of rehabilitation policies (19, 20). These results suggest that reducing the duration of rehabilitation may be an important strategy for controlling medical expenses (21, 22). Nevertheless, existing evidence indicates that early rehabilitation can effectively reduce the incidence of complications and promote functional recovery (23), while also shortening treatment duration (24, 25), and lowering medical expenses (26, 27). Beyond physical recovery, early rehabilitation may help alleviate psychological distress, such as depression and anxiety (28, 29), enhance confidence in returning to work and social reintegration (30, 31). Ultimately, these effects could contribute to reduced expenditures from the occupational injury insurance fund. Therefore, standardizing early rehabilitation as a routine practice holds clear practical and policy significance (32).

Regarding individual factors, the study found that male sex and the 60–69 age group significantly affected the duration of rehabilitation, whereas they did not significantly influence the timing of rehabilitation initiation. Specifically, male workers with occupational injuries had significantly shorter duration of rehabilitation than female workers at the 25th and 50th percentiles. This difference remained statistically significant after controlling for potential confounding variables, including the injury site, surgical status, and the administrative origin of injury certification. These findings suggest that sex may represent an important independent factor influencing rehabilitation progress. Female workers with occupational injuries may encounter greater physiological challenges and limitations in social support throughout the rehabilitation process. This finding is consistent with previous studies suggesting that women tend to have higher postoperative pain sensitivity and slower recovery of muscle strength, physiological differences that may partly account for their slower rehabilitation progress (33). In the descriptive analysis, workers aged 60–69 years appeared to have longer the timing of rehabilitation initiation and extended the duration of rehabilitation. However, after adjustment for potential confounders, this age group was associated with a significantly shorter the duration of rehabilitation across all quantiles, while no consistent association was observed for the timing of rehabilitation initiation. This discrepancy suggests that the prolonged the duration of rehabilitation observed in the unadjusted analysis may reflect the influence of other factors rather than age itself. Possible explanations include greater injury severity, delayed access to rehabilitation services, and limited awareness or utilization of rehabilitation policies among older workers, which may particularly affect the timing of rehabilitation initiation. Previous studies have indicated that older individuals with occupational injuries often have reduced access to rehabilitation information and resources, which may contribute to delay the timing of rehabilitation initiation and prolonged the duration of rehabilitation in unadjusted analyses (34).

Regarding surgical factors, the study results show that workers with occupational injuries who underwent surgery experienced significantly longer rehabilitation periods compared to those who did not have surgery. The timing of rehabilitation intervention was also notably delayed, indicating a postponement in post-surgical rehabilitation. Although surgery is a vital treatment for severe injuries, the absence of timely post-surgical rehabilitation can lead to further worsening of functional impairments (35), extend the duration of rehabilitation, and increase medical and return-to-work costs (36, 37). Therefore, it is crucial to strengthen systematic management of early postoperative rehabilitation, promote coordinated planning of rehabilitation interventions with medical treatment, and improve overall rehabilitation efficiency to decrease societal and individual costs.

This study found that limb injuries were the most common occupational injuries among workers in Yancheng. Injury site was significantly associated with the duration of rehabilitation (*p* < 0.001), although there were no significant differences in the timing of rehabilitation initiation across injury sites (*p* > 0.05). Quantile regression analysis indicated that the effect of injury site on the duration of rehabilitation was most pronounced at the lower and median quantiles, while it diminished at the upper quantile, suggesting that prolonged rehabilitation periods may be influenced by factors such as injury severity, complications, or individual adherence, among others. Notably, hand injuries accounted for a large portion of cases, and their duration of rehabilitation was significantly different from those of head and neck, upper limb, and lower limb injuries, consistent with findings from previous studies (38, 39). The hands are essential for daily activities and work tasks, and play a crucial role in psychological and social recovery after an injury. Hand injuries not only affect work ability and cause financial hardship for families but also contribute to broader socioeconomic issues (40, 41). Evidence suggests that early rehabilitation interventions can significantly enhance hand function (42, 43), decrease medical costs (44), and reduce time away from work for workers with occupational injuries (45). The results of this study indicate that the median timing of rehabilitation intervention for employees with work-related burn injuries receiving treatment exceeded 150 days, while the shortest median duration of rehabilitation was 30 days. A statistically significant disparity was observed in rehabilitation duration when compared to that of workers with head and neck injuries (*p* < 0.001), suggesting variations in injury complexity or treatment needs. The observed pattern of delayed rehabilitation initiation and shorter rehabilitation duration among burn patients in Yancheng indicates that rehabilitation often begins only after the wound has fully healed (46, 47). The findings suggest that the delayed rehabilitation initiation for burn injuries in Yancheng may reflect inadequate resource allocation and insufficient recognition of the importance of early rehabilitation in burn treatment. Postponed rehabilitation in such cases is frequently linked to adverse outcomes. These include hypertrophic scarring, joint contractures, and psychological sequelae such as depression and anxiety (48). Such outcomes can negatively affect both quality of life and the ability to return-to-work (49, 50). Evidence from existing literature consistently highlights the benefits of early, structured, and multidisciplinary rehabilitation interventions in improving long-term functional and psychosocial recovery in burn patients (51, 52). Evidence from existing literature consistently highlights the benefits of early, structured, and multidisciplinary rehabilitation interventions in improving long-term functional and psychosocial recovery in burn patients (53). Head and neck injuries are often associated with conditions such as traumatic brain injury or motor impairments, whereas multiple-site injuries frequently involve damage to two or more functional regions. These complex injury types are more challenging to treat and typically require longer recovery times to regain function (20).

Overall, the median timing of rehabilitation initiation among workers with occupational injuries in Yancheng was 126 days, while the median duration of rehabilitation was 30 days. The timing of rehabilitation initiation appears substantially delayed compared with typical clinical practice, where rehabilitation is generally recommended to commence early following surgery or acute management (54). In contrast, the duration of rehabilitation also appears relatively prolonged when compared with reports from other injury populations (55). This finding suggests that both the delayed timing of rehabilitation initiation and the extended duration of rehabilitation may be influenced by multiple factors. The delayed timing of rehabilitation initiation may be associated with administrative processes related to occupational injury certification, referral procedures, and access to designated rehabilitation services. Meanwhile, the prolonged duration of rehabilitation may reflect delayed initiation, variability in injury severity, as well as differences in rehabilitation service delivery and resource allocation. Most injured workers receiving rehabilitation were male and aged 40–59 years. This aligns with previous studies (7), indicating that middle-aged male workers are more likely to be employed in physically demanding and high-risk occupations. Sociocultural roles and individual characteristics may contribute to this pattern (56). These results highlight young and middle-aged male workers as a key population for preventing occupational injuries. Targeted measures should focus on standardized professional training and increasing awareness of rehabilitation policies among this group (57).

## 5 Limitations

However, several limitations warrant consideration. First, this retrospective study was conducted using data from a single occupational rehabilitation center in Yancheng, which may restrict the generalizability of the findings to other regions and healthcare settings. Second, the analysis included only individuals who had already initiated rehabilitation, potentially excluding those with delayed access or no access to rehabilitation services, thereby introducing selection bias. Third, some key variables were not available, including injury severity, socioeconomic characteristics, workplace-related factors, and employer involvement. In addition, the analysis was limited to physical injuries, without consideration of mental health conditions, and lacked detailed job title information. The absence of standardized injury classification (e.g., ICD codes) may also have reduced the accuracy of injury categorization. Together, these factors may have influenced the interpretation of both the timing of rehabilitation initiation and the duration of rehabilitation. Further research using prospective, multicenter designs with more comprehensive data collection is needed to strengthen the evidence base and improve the generalizability of the findings.

## 6 Conclusion

In Yancheng, workers with occupational injuries were mainly young and middle-aged males, with limb injuries being the most common. The significant variation in the timing of rehabilitation initiation across administrative regions may be attributable to differences in regional healthcare practices or policy implementation. Meanwhile, the duration of rehabilitation was influenced by various factors, including sex, age, surgical status, and the location of the injury. The findings indicate that a more evidence-based, standardized, and regionally integrated rehabilitation system could contribute to improved efficiency and equitable access to services in Yancheng.

To address these challenges, collaboration among the government, employers, and designated rehabilitation institutions is necessary. From a policy standpoint, the government should focus on increasing public and stakeholder awareness of occupational rehabilitation policies, simplifying the rehabilitation referral process, and strengthening adherence to the principle of “rehabilitation prior to disability assessment.” Assessment” (58). At the enterprise level, employers may support these efforts by facilitating timely access to rehabilitation services and maintaining safe working environments for high-risk groups (1). Designated rehabilitation institutions, in turn, should focus on enhancing workforce capacity through injury-specific training, ensuring proper allocation of rehabilitation resources, and providing high-quality, personalized rehabilitation treatments. Additionally, vocational rehabilitation programs should be developed to boost return-to-work rates.

A cross-sector collaborative framework is crucial for establishing a standardized, efficient, and well-coordinated occupational rehabilitation system. Establishing an integrated system may facilitate timely access to rehabilitation services and contribute to improved recovery outcomes for workers with occupational injuries in Yancheng.

## Data Availability

The original contributions presented in the study are included in the article/supplementary material, further inquiries can be directed to the corresponding authors.

## References

[1] Yosef T Sineshaw E Shifera N. Occupational injuries and contributing factors among industry park construction workers in Northwest Ethiopia. Front Public Health. (2022) 10:1060755. doi: 10.3389/fpubh.2022.1060755 36703838 PMC9872008

[2] Frank P Halim H Bálint N C MN. Global, regional and national burden of disease attributable to 19 selected occupational risk factors for 183 countries, 2000–2016: a systematic analysis from the WHO/ILO joint estimates of the work-related burden of disease and injury. Scand J Work Environ Health. (2022) 48:158–68. doi: 10.5271/sjweh.4001 34806754 PMC9045235

[3] Chen YQ Han Y Chen G. Analysis of the current situation of work-related injuries in Kunshan City from 2016 to 2021. Chin Occup Med. (2024) 51:223–8. doi: 10.20001/j.issn.2095-2619.20240419

[4] Mari Nilsen S Rikke Helene M Thomas J Peter Solvoll L Kjersti D Idun E . Work ability in the year after rehabilitation-results from the RehabNytte cohort. J Clin Med. (2023) 12:7391. doi: 10.3390/brainsci16010073 38068445 PMC10707470

[5] Jarna P Arto L. How can social insurers promote return to work in occupational rehabilitation? A quantitative, cross-sectional study. BMC Public Health. (2021) 21:1687. doi: 10.1186/s12889-021-11758-w 34530777 PMC8444422

[6] Aasdahl L Fimland MS Bjørnelv GMW Gismervik S Johnsen R Vasseljen O . Economic evaluation of inpatient multimodal occupational rehabilitation vs. outpatient acceptance and commitment therapy for sick-listed workers with musculoskeletal- or common mental disorders. J Occup Rehabil. (2023) 33:463–72. doi: 10.1007/s10926-022-10085-0, 36949254 PMC10495483

[7] Alamneh YM Wondifraw AZ Negesse A Ketema DB Akalu TY. The prevalence of occupational injury and its associated factors in Ethiopia: a systematic review and meta-analysis. J Occup Med Toxicol. (2020) 15:14. doi: 10.1186/s12995-020-00265-0, 32518580 PMC7271426

[8] Alexandra L Marie-Ève M Valérie L Claude V Marie-Ève L Andrée-Anne D. Integrative prevention at work: a concept analysis and meta-narrative review. J Occup Rehabil. (2022) 33:301–15. doi: 10.1007/s10926-022-10073-4 36348235 PMC9643891

[9] Mei Ling T Elliot E Benjamin Wei DY Wei Xiang E Su Xian T John Wah L . A hospital-based return-to-work programme in Singapore. Ind Health. (2022) 61:269–74. doi: 10.2486/indhealth.2022-0072 35584948 PMC10398161

[10] Chia-Lin Y Yan-Ru Y Chuan-Man C Pei-Ling T. Does category of strength predict return-to-work after occupational injury? BMC Public Health. (2022) 22:1472. doi: 10.1186/s12889-022-13817-2 35918669 PMC9344704

[11] Miriam M Nina G Markus B Matthias B. Work-related medical rehabilitation in patients with mental disorders: the protocol of a randomized controlled trial (WMR-P, DRKS00023175). BMC Psychiatry. (2021) 21:225. doi: 10.1186/s12888-021-03181-7 33941123 PMC8091693

[12] Phillip R Kathy S-J Johnny WC Sara M. Retain Kentucky: a return-to-work and stay-at-work program for people with disabilities grounded in the conservation of resources theory. Work. (2022) 72:3–8. doi: 10.3233/WOR-223633 35491810

[13] Nahal S Bryan G Amin S. State-federal vocational rehabilitation services, demographic characteristics and employment outcomes for native Americans with mental illnesses. Community Ment Health J. (2023) 60:442–56. doi: 10.1007/s10597-023-01191-1 37828363

[14] Zhou YB Mi ZX. Analysis of the development status quo and countermeasure research of work injury rehabilitation hospitals in the new era: taking Beijing as an example. Chin Hosp. (2023) 27:43–6. doi: 10.19660/j.issn.1671-0592.2023.12.11

[15] Glaesener JJ Simmel S. Trauma rehabilitation: implementation and challenges. Unfallchirurgie (Heidelb). (2023) 126:26–33. doi: 10.1007/s00113-022-01259-9, 36416891

[16] Wang Y Zhai H Feng Y Zhang Y Peng JP. The study of early rehabilitation intervention on industrial injury at the identification stage. China Acad J Electron Publ House. (2015) 4:58–60. doi: 10.3969/j.issn.1674-3830.2015.04.024

[17] Feng Y Wang Y Zhai H. Exploration and thinking on the early intervention of the rehabilitation of industrial injury. China Acad J Electron Publ House. (2018) 9:59–61,5. doi: 10.19546/j.issn.1674-3830.2018.9.013

[18] Feng Y Wang Y Yang LY Zhai H. Exploration and practice of industrial injury rehabilitation in Shanghai. China Acad J Electron Publ House. (2020) 1:68–71. doi: 10.19546/j.issn.1674-3830.2020.1.014

[19] Zhou YB Zhang Q Yin M Chi S Gui ZL Guo YJ . Analysis of influencing factors on insurance settlement of inpatient expenses for four rehabilitation disease types. Chin Hosp. (2024) 28:56–8.

[20] de Munter L Geraerds A de Jongh MAC van der Vlegel M Steyerberg EW Haagsma JA . Prognostic factors for medical and productivity costs, and return to work after trauma. PLoS One. (2020) 15:e0230641. doi: 10.1371/journal.pone.0230641 32210472 PMC7094860

[21] Malekzadeh H Golpayegani M Ghodsi Z Sadeghi-Naini M Asgardoon M Baigi V . Direct cost of illness for spinal cord injury: a systematic review. Global Spine J. (2022) 12:1267–81. doi: 10.1177/21925682211031190, 34289308 PMC9210246

[22] Jiang N Wei N L ZY Zhang Q Yi XZ Chen WQ . Influential factors of average hospitalization costs of rehabilitation inpatients based on quantile regression model. Chin J Rehabil Theory Pract. (2024) 30:397–403. doi: 10.3969/j.issn.1006-9771.2024.04.004

[23] Voss MR Homa JK Singh M Seidl JA Griffitt WE. Outcomes of an interdisciplinary work rehabilitation program. Work. (2019) 64:507–14. doi: 10.3233/WOR-193012, 31658084 PMC7029322

[24] Mekonnen TH Sheehan LR Di Donato M Collie A Russell G. Relationship between the timing of physical therapy commencement and the duration of work disability: a retrospective cohort analysis of work-related low back pain claims. BMC Public Health. (2025) 25:1329. doi: 10.1186/s12889-025-22574-x, 40205570 PMC11983916

[25] Zhao X Gong KT. Effect of early intervention of occupational therapy on functional recovery of patients after replantation of severed fingers. Chin J Hand Surg. (2023) 39:136–9. doi: 10.3760/cma.j.cn311653-20220624-00176

[26] Al Qadire M Abdelrahman H. Early rehabilitation of patients in the ICU may reduce long-term healthcare costs. Evid Based Nurs. (2023) 27:ebnurs-2023-103759. doi: 10.1136/ebnurs-2023-103759, 37286308

[27] Zhu XP Xu H Wang L Wang J Zhang H. Relationship of preoperative sleep quality and early rehabilitation after unicompartmental knee arthroplasty. Zhongguo Zuzhi Gongcheng Yanjiu. (2023) 27:5806–11. doi: 10.12307/2023.706

[28] Dhanaraj I Rajaratnam V Jaafar H Morgan K. The psychological impact of hand injuries among foreign Workers in Singapore. Cureus. (2024) 16:e60772. doi: 10.7759/cureus.60772, 38903327 PMC11188967

[29] Feng Y Guo J Wang Y Wang HF Huo H. Effects of early rehabilitation on expectation and perception for inpatients with work injury. Chin J Rehabil Theory Pract. (2019) 25:357–62. doi: 10.19660/j.issn.1671-0592.2023.12.11

[30] Bae SW Lee MY Park SW Lee G Leigh JH. Satisfying medical and rehabilitation needs positively influences returning to work after a work-related injury: an analysis of national panel data from 2018 to 2019. BMC Public Health. (2021) 21:2017. doi: 10.1186/s12889-021-12064-1, 34740350 PMC8571869

[31] Dong J Sun FL Wang HQ Lin F Liu ZY Li K . Influence of course of disease before rehabilitation intervention on mental stateand employment willingness of industrial injury patients with limb fractures. Chin J Rehabil Med. (2019) 34:351–4. doi: 10.3870/zgkf.2019.07.004

[32] Dorstyn D Oxlad M Whitburn S Fedoric B Roberts R Chur-Hansen A. Client and staff perspectives regarding effective work injury rehabilitation. Aust Health Rev. (2023) 47:339–43. doi: 10.1071/AH22256, 36921621

[33] Bartley EJ Fillingim RB. Sex differences in pain: a brief review of clinical and experimental findings. Br J Anaesth (2013) 111:52–8. doi: 10.1093/bja/aet127 23794645 PMC3690315

[34] Feng Y Yang LY Wu CG Zhang CP Wei M Shao YQ . Status quo and influencing factors of rehabilitation intention among injured workers in Shanghai, China. J Tongji Univ (Med Sci). (2022) 43:711–7. doi: 10.12289/j.issn.1008-0392.21494

[35] Lv HZ Hou ZY Wang J Li J Zheng ZL Lian XD . Factor related to functional recovery of the knee following tibial plateau fracture complicated with intercondyar ridgy fracture. Chin J Orthop Trauma. (2021) 23:132–7. doi: 10.3760/cma.j.cn115530-20200922-00618

[36] Groot L Latijnhouwers DAJM Reijman M Verdegaal SHM Vliet Vlieland TPM Gademan MGJ. Recovery and the use of postoperative physical therapy after total hip or knee replacement. BMC Musculoskelet Disord. (2022) 23:666. doi: 10.1186/s12891-022-05429-z, 35831841 PMC9277921

[37] Myles PS. More than just morbidity and mortality - quality of recovery and long-term functional recovery after surgery. Anaesthesia. (2020) 75:e143-e50. doi: 10.1111/anae.14786 31903564

[38] Zhao J Guo W Zeng X Kan S. Research progress of early postoperative rehabilitation for acute Achilles tendon rupture after surgical repair. Zhongguo Xiu Fu Chong Jian Wai Ke Za Zhi. (2019) 33:382–6. doi: 10.7507/1002-1892.201807146, 30874399 PMC8337926

[39] Fu CD Lin DY Qiu XL Sun SH Feng Q Wang HW . Analysis the risk factors of disabling occupational hand injuries and the preventive measures. Ind Hlth Occup Dis. (2020) 46:444–6. doi: 10.13692/j.cnki.gywsyzyb.2020.06.002

[40] Glen LZQ Wong JYS Tay WX Weng J Cox G Cheah AEJ. Forecasting the rate of hand injuries in Singapore. J Occup Med Toxicol. (2022) 17:9. doi: 10.1186/s12995-022-00350-6, 35509052 PMC9066836

[41] Chen SQ Wang Y Shi CH Feng Y Wu SB. Hand injuries hospitalized patients medical economic burden survey and influence factors analysis. Chin J Ind Hyg Occup Dis. (2017) 35:451–4. doi: 10.3760/cma.j.issn.1001-9391.2017.06.013 28780824

[42] Chen YY Yang CS Lee BO. Illness perceptions, disabilities, and quality of life of patients with hand injury: a prospective study. J Trauma Nurs. (2021) 28:90–9. doi: 10.1097/jtn.0000000000000566, 33667203

[43] Huang WZ Yan W Wang ZJ Cui SY Mai GH Li YF . Effect of work injuries rehabilitation investigate system on early intervention of hand trauma rehabilitation. Zhongguo Kangfu Lilun Yu Shijian. (2017) 23:1226–30. doi: 10.3969/j.issn.1006-9771.2017.10.021

[44] Lalchandani GR Halvorson RT Zhang AL Lattanza LL Immerman I. Patient outcomes and costs after isolated flexor tendon repairs of the hand. J Hand Ther. (2022) 35:590–6. doi: 10.1016/j.jht.2021.04.015, 34016517

[45] Lu XW Gao JZ Huang R Sun TB Gao CS. A validity study on predicting return to work after occupational hand trauma. Chin J Rehabil Med. (2019) 34:678–82. doi: 10.3969/j.issn.1001-1242.2019.06.010

[46] Zeng Z Li N Yang L Feng X Zuo F Luo G . Cost analysis of severe burn victims in Southwest China: a 7-year retrospective study. Front Public Health. (2022) 10:1052293. doi: 10.3389/fpubh.2022.1052293, 36699890 PMC9868295

[47] Rivas E Foster J Crandall CG Finnerty CC Suman-Vejas OE. Key exercise concepts in the rehabilitation from severe burns. Phys Med Rehabil Clin N Am. (2023) 34:811–24. doi: 10.1016/j.pmr.2023.05.003, 37806699 PMC10731385

[48] Zaman NI Zahra K Yusuf S Khan MA. Resilience and psychological distress among burn survivors. Burns. (2023) 49:670–7. doi: 10.1016/j.burns.2022.05.001, 35842271

[49] Spronk I Van Loey NEE van der Vlies CH Haagsma JA Polinder S van Baar ME. Activity impairment, work status, and work productivity loss in adults 5-7 years after burn injuries. J Burn Care Res. (2022) 43:256–62. doi: 10.1093/jbcr/irab047, 33693704 PMC8737115

[50] Tian YF Liu Y. Research advances on functional training robots in burn rehabilitation. Chin J Burns Wounds. (2022) 38:580–4. doi: 10.3760/cma.j.cn501120-20210416-00131 PMC11705246 35764586

[51] Kara S Seyhan N Öksüz S. Effectiveness of early rehabilitation in hand burns. Ulus Travma Acil Cerrahi Derg. (2023) 29:691–7. doi: 10.14744/tjtes.2023.22780, 37278077 PMC10315933

[52] Schieffelers DR Ru T Dai H Ye Z van Breda E Van Daele U . Effects of early exercise training following severe burn injury: a randomized controlled trial. Burns Trauma. (2024) 12:tkae005. doi: 10.1093/burnst/tkae005, 38716050 PMC11075770

[53] Fure SCR Howe EI Andelic N Brunborg C Sveen U Røe C . Cognitive and vocational rehabilitation after mild-to-moderate traumatic brain injury: a randomised controlled trial. Ann Phys Rehabil Med. (2021) 64:101538. doi: 10.1016/j.rehab.2021.101538, 33957293

[54] Fan MC Li SF Sun P Bai GT Wang N Han C . Early intensive rehabilitation for patients with traumatic brain injury: a prospective pilot trial. World Neurosurg. (2020) 137:e183–8. doi: 10.1016/j.wneu.2020.01.113, 32001397

[55] Calderone A Bonanno L Rifici C Calabrò RS. Early functional recovery trajectories after severe traumatic brain injury: a secondary analysis of the TBIMS National Database. Brain Sci. (2026) 16:73. doi: 10.3390/brainsci16010073, 41594794 PMC12838949

[56] Biswas A Harbin S Irvin E Johnston H Begum M Tiong M . Differences between men and women in their risk of work injury and disability: a systematic review. Am J Ind Med. (2022) 65:576–88. doi: 10.1002/ajim.23364, 35578160 PMC9321824

[57] Prasetyo YT Garcia MM Dewi RS Chuenyindee T Kurata YB Widia M. Accident patterns and prevention measures for occupational injuries in the Philippine food and beverage manufacturing industry. Work. (2022) 73:1307–24. doi: 10.3233/WOR-210662, 36057804

[58] Zhou YB Zhang Q Yin M Chi S Gui ZL Guo YJ . Analysis of infuencing factors on insurance settlement of inpatient expenses for four rehabilitation disease types. Chinese Hosp. (2023) 27:43–6. doi: 10.19660/j.issn.1671-0592.2023.12.11

